# Supplementary material for: Characterization of genetic diversity and population structure within Staphylococcus chromogenes by multilocus sequence typing
Source: PLoS One. 2021 Mar 15;16(3):e0243688. doi: 10.1371/journal.pone.0243688 (PMC7959370; doi:10.1371/journal.pone.0243688)
Supplement: S2 Table — (DOCX) [file pone.0243688.s002.docx]

**S2 Table. Genetic loci and primer sequences used in MLST scheme**

| **Locus** | **Contig** | **Locus Tag** | **Gene** | **PCR Primers (5’→3’)** | **PCR Product (bp)** | **Gene sequence segment used for MLST (length)** |
| --- | --- | --- | --- | --- | --- | --- |
| *arcC* | 11 | SCHR_RS09835 | Carbamate kinase | F: CGGCGATTCGACAAACACTC  R: TGGCAACATCGACCCTTCTG | 746 | 133 - 720 (588 bp) |
| *hutU* | 4 | SCHR_RS06485 | Urocanate hydrase | F: AAGGGGTTGTCATCGGTGTA  R: GCATCGGAACCGTCTTTCAT | 829 | 616 – 1308 (693 bp) |
| *fumC* | 16 | SCHR_RS10910 | Fumerate hydratase | F: TGCATGTCGCACTATATCAC  R: CATCAATATGTTCCTCAATCG | 756 | 496 – 1131 (636 bp) |
| *dnaJ* | 3 | SCHR_RS04660 | chaperone protein  dnaJ | F: AAAGGGAGCGATAGCATTGG  R: CATCACCTAACGCAGCTTGT | 869 | 46 – 792 (747 bp) |
| *glpF* | 2 | SCHR_RS03485 | glycerol uptake  facilitator | F: TACGGTTAGGCAAGGAGTCT  R: AACGACCTTGGTAGGCCAAT | 759 | 25 – 636 (612 bp) |
| *menF* | 1 | SCHR_RS00550 | Isochorismate  synthase | F: TGTCACACCTGAAGAACAACA  R: TAACGCTTGGTTACCTTGAATC | 730 | 592 – 1188 (597 bp) |
| *pta* | 1 | SCHR_RS02430 | Phosphate acetyl  transferase | F: AACGCCCCCTTGGAAAAGTC  R: TGGATTTTAGCGCCCGGTG | 870 | 13 – 702 (690 bp) |
